# Supplementary material for: Biosynthesis of Phenylamide Phytoalexins in Pathogen-Infected Barley
Source: Int J Mol Sci. 2019 Nov 6;20(22):5541. doi: 10.3390/ijms20225541 (PMC6888128; doi:10.3390/ijms20225541)
Supplement: Supplementary file 1 [file ijms-20-05541-s001.pdf]

Supplementary materials

Biosynthesis of Phenylamide Phytoalexins in Pathogen-Infected Barley

Naoki Ube, Yukinori Yabuta, Takuji Tohnooka, Kotomi Ueno, Shin Taketa, Atsushi Ishihara*

E-mail address: aishihara@tottori-u.ac.jp (A. Ishihara).

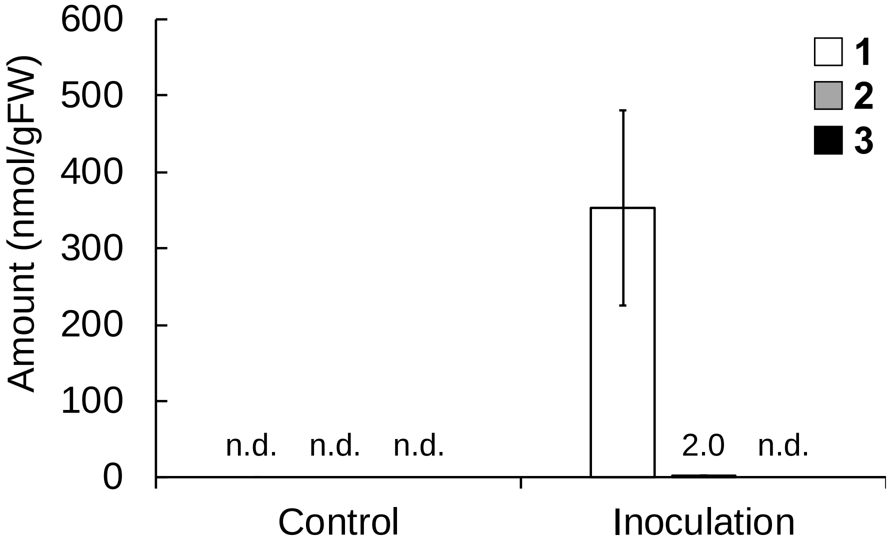

Supplementary Figure S1. Accumulation of compounds **1**–**3** in *Fusarium culmorum*-infected wheat roots at 72 h after inoculation. Values and error bars represent mean ± SD (n = 3). n.d., not detected.

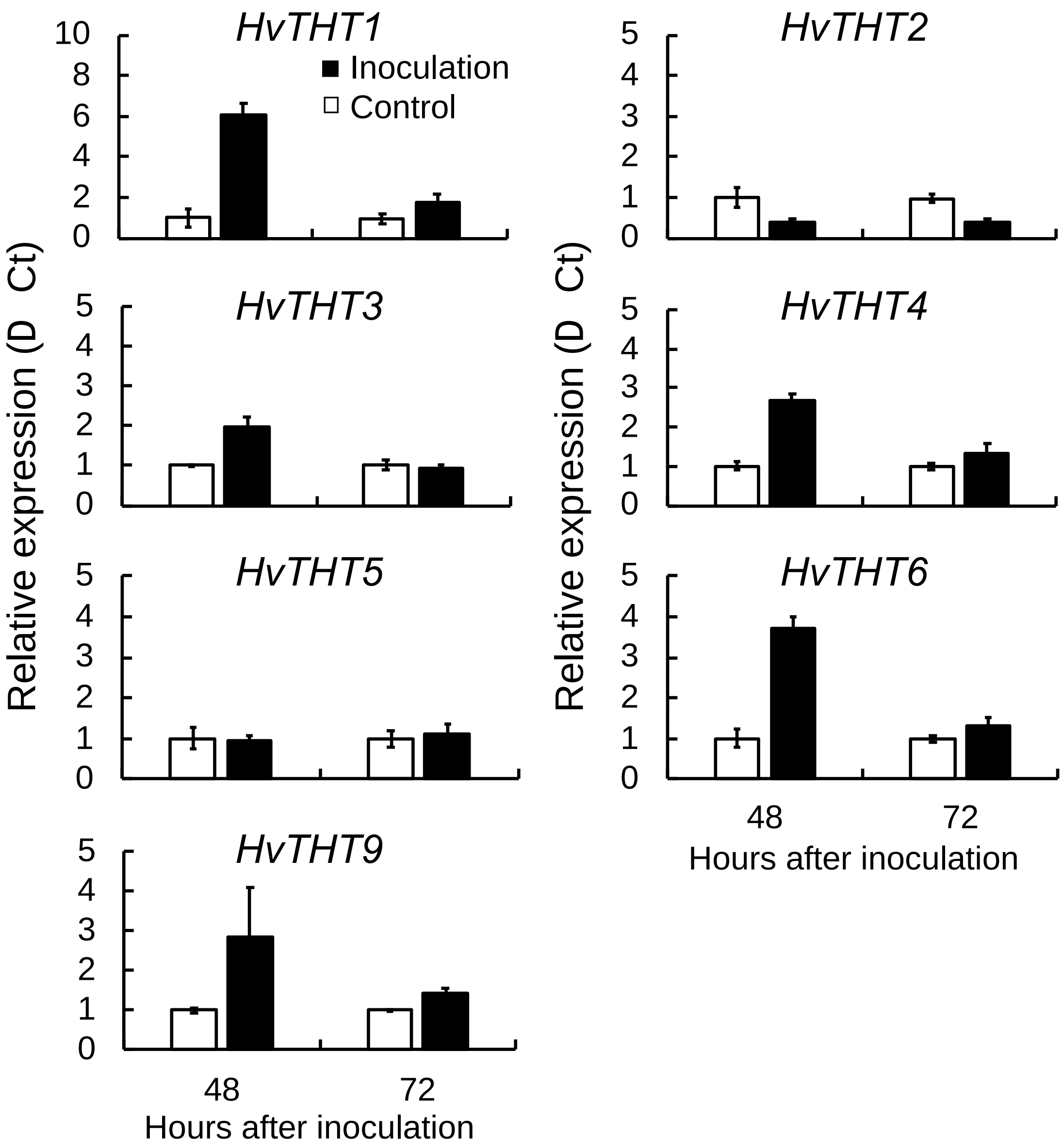

Supplementary Figure S2. Effects of *Fusarium culmorum* infection on the expression of *HvTHT* genes in barley roots. Total RNA were extracted 48 h and 72 h after inoculation. expression levels were normalized using the ADP-ribosylation factor-like protein (*ADP*) gene as an inner control and are expressed as relative values compared to those of control roots and leaves. Values and error bars represent mean ± SD (*n* = 3).

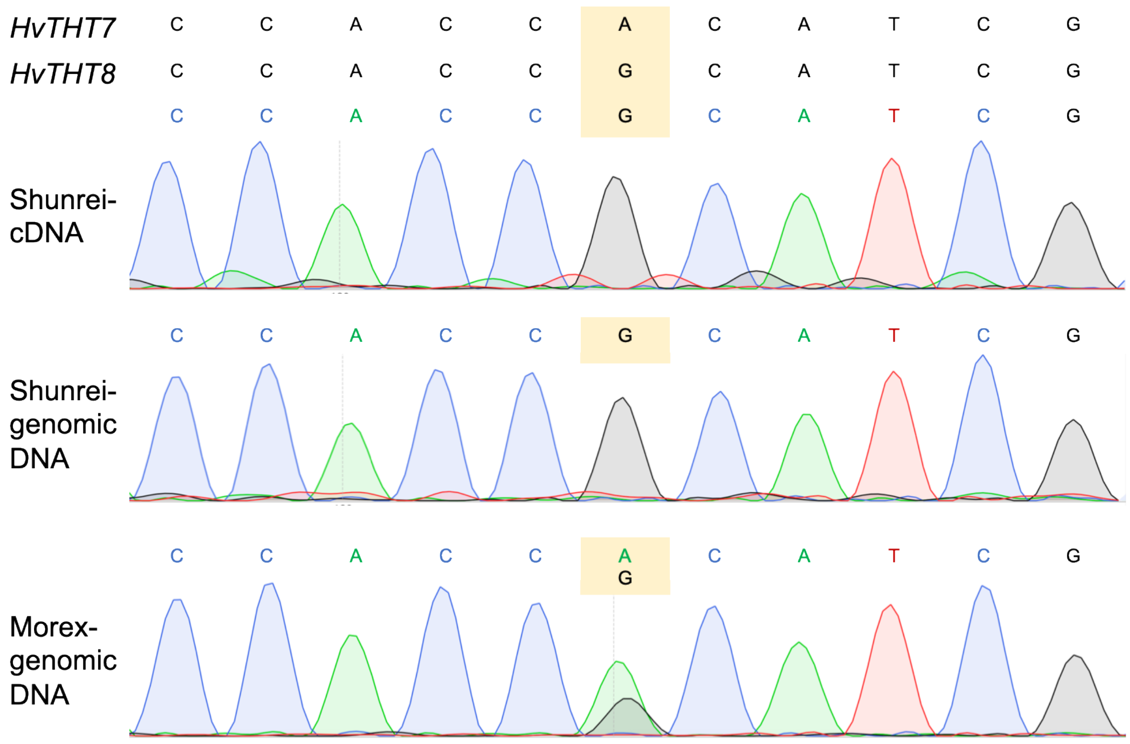

Supplementary Figure S3. Sequence analyses of *HvTHT7/8* fragments amplified from cDNA of *F. culmorum*-infected barley ‘Shunrei’ roots and from the genomic DNA of ‘Shunrei’ and ‘Morex’. The green, red, black, and blue lines indicated A, T, G, and C, respectively. The letters above the waves indicated nucleotides around the single nucleotide substitution site of *HvTHT7/8* (490–500 bp from the start codon).

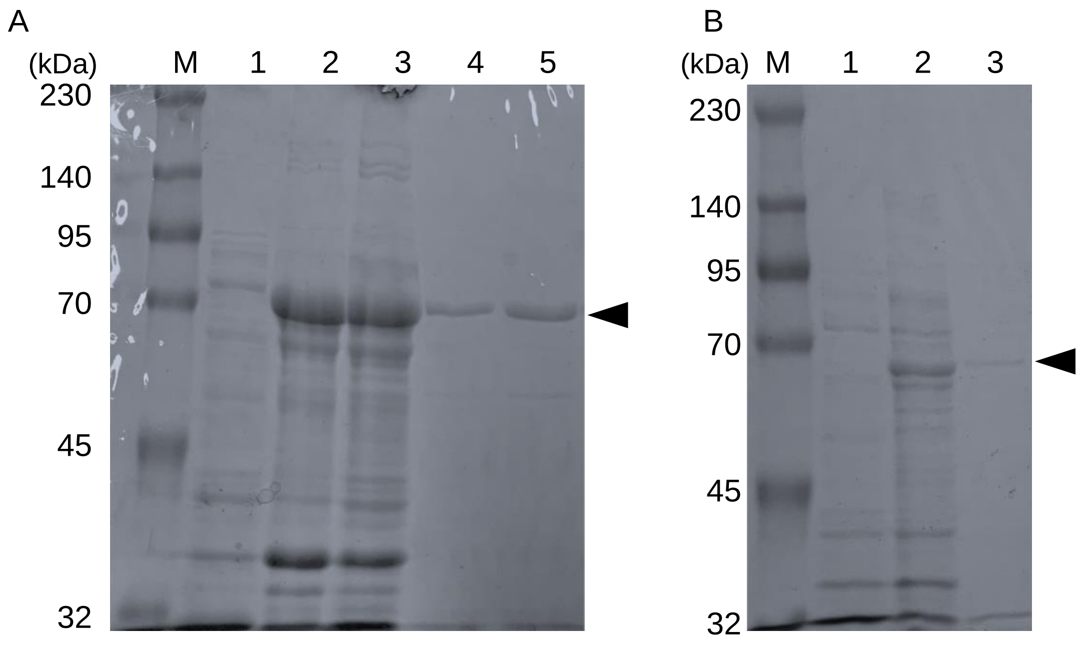

Supplementary Figure S4. SDS-PAGE analysis of recombinant HvTHT2, HvTHT7 and HvTHT8. Proteins from each purification step were separated by SDS-PAGE on an 8.5% gel and stained with Coomassie Brilliant Blue R-250. (A) Lane M, Molecular marker; Lane 1, crude *E. coli* extract; Lane 2, crude extract of *HvTHT7*-expressing *E. coli*; Lane 3, crude extract of *HvTHT8*-expressing *E. coli*; Lane 4, HvTHT7 purified by metal-affinity chromatography; Lane 5, HvTHT8 purified by metal-affinity chromatography. (B) Lane M, Molecular marker; Lane 1, crude *E. coli* extract; Lane 2, crude extract of HvTHT2-expressing *E. coli*; Lane 3, HvTHT2 purified by metal-affinity chromatography. Arrowheads indicate HvTHT proteins.

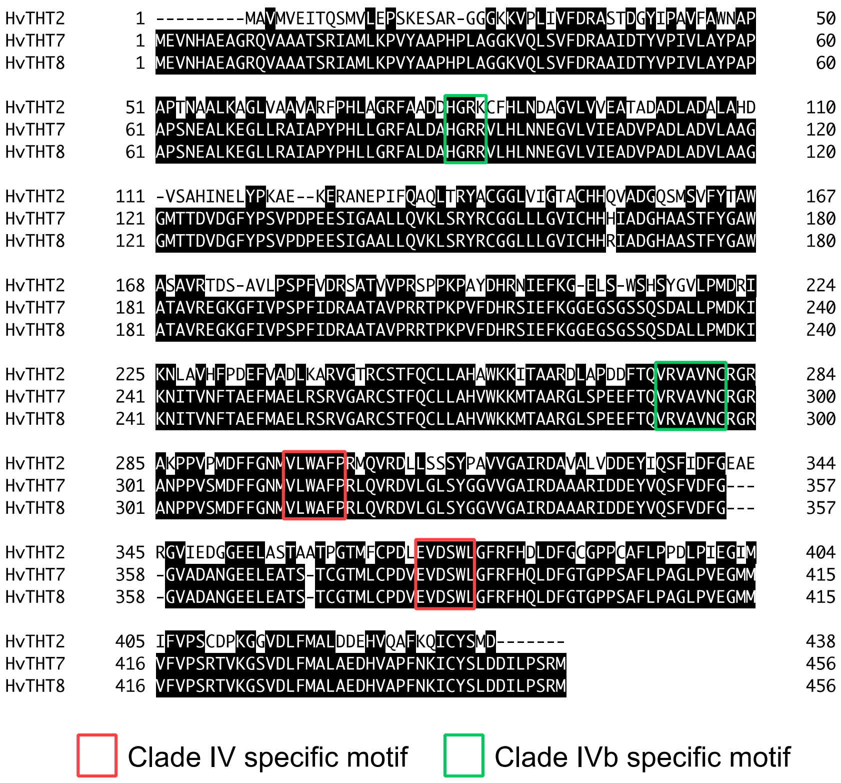

Supplementary Figure S5. Amino acid sequences of HvTHT2, HvTHT7, and HvTHT8. The red and green squares indicate clade IV and IVb specific motifs, respectively. The HvTHT amino-acid sequences were obtained from the EnsemblPlants database (http://plants.ensembl.org/Hordeum_vulgare/Info/Index?db=core).

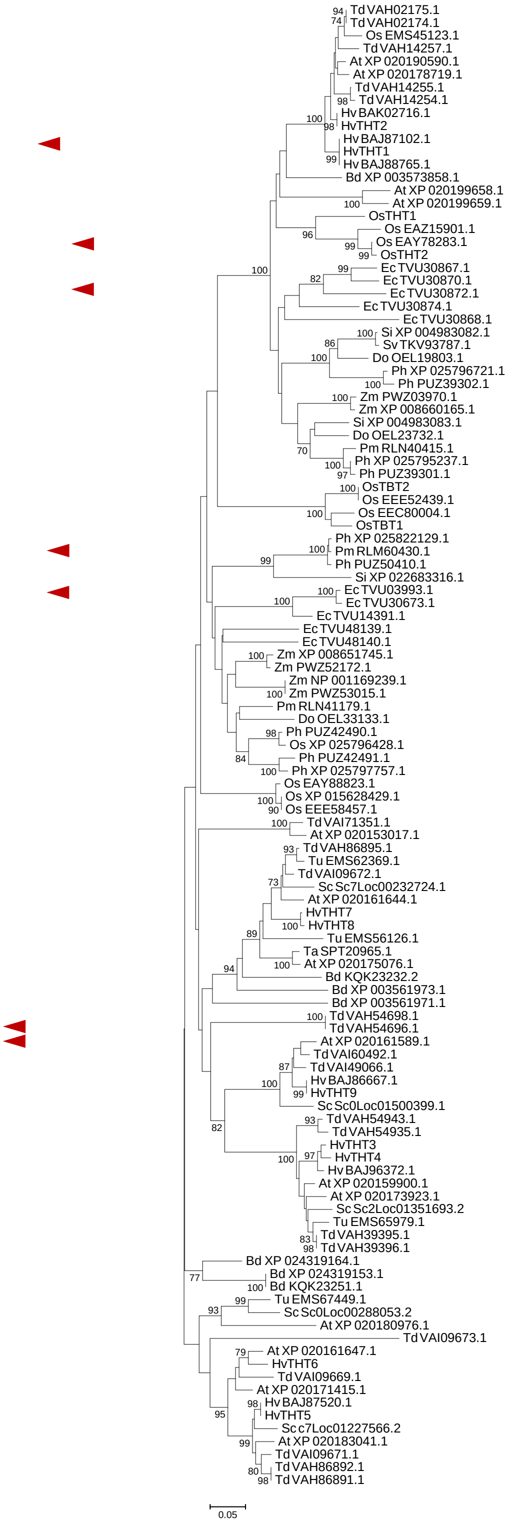

Supplementary Figure S6. Relationships between HvTHT proteins and HvTHT like proteins from various species. A dendrogram was generated form sequences of 115 HvTHT like proteins. Bootstrap values >70% (based on 1,000 replications) are indicated at each node (bar = 0.05 amino acid substitutions per site). Protein sequences

were obtained from GenBank (https://blast.ncbi.nlm.nih.gov/Blast.cgi) and IPK Rye Blast Server (https://webblast.ipk-gatersleben.de/ryeselect/). Abbreviation of species before the accession number were as follows: At (*Aegilops tauschii*), Bd (*Brachypodium distachion*), Do (*Dichanthelium oligosanthes*), Ec (*Eragrostis curvula*), Hv (*Hordeum vulgare*), Os (*Oryza sativa*), Ph (*Panicum hallii*), Pm (*Panicum miliaceum*), Sc (*Secale cereale*), Si (*Setaria italica*), Sv (*Setaria viridis*), Ta (*Tirticum aestivum*), Td (*Triticum durum*), Tu (*Triticum urartu*), Zm (*Zea mays*). Accessino numer of OsTHTs OsTBTs were as follow: OsTHT1 (XP_015613139.1), OsTHT2 (XP_015612968.1), OsTBT1 (XP_015615935.1), and OsTBT2 (XP_015615816.1). The HvTHT amino-acid sequences were obtained from the EnsemblPlants database (http://plants.ensembl.org/Hordeum_vulgare/Info/Index?db=core). Red arrowheads indicate HvTHT2, HvTHT7, HvTHT8, OsTHT1, OsTHT2, OsTBT1, and OsTBT2.

Supplementary table S1. *HvTHT* genes detected by a search on the database, EnsemblPlants (http://plants.ensembl.org/Hordeum_vulgare/Info/Index?db=core).

Gene	Gene code	Similarity of amino acid sequnece (%)			
		OsTBT1	OsTBT2	OsTHT1	OsTHT2
HvTHT1	HORVU1Hr1G019380	78.7	77.7	86.3	87.1
HvTHT2	HORVU1Hr1G019410.1	78.5	78.1	85.9	87.0
HvTHT3	HORVU2Hr1G125270.1	81.2	80.5	78.4	78.6
HvTHT4	HORVU2Hr1G125370.1	80.9	80.4	79.1	79.3
HvTHT5	HORVU4Hr1G077680.11	73.9	73.1	72.7	73.6
HvTHT6	HORVU4Hr1G077720.8	80.8	80.4	80.1	81.0
HvTHT7	HORVU4Hr1G077780.1	80.7	80.0	77.9	79.5
HvTHT8	HORVU4Hr1G077790	80.6	79.9	77.9	79.5
HvTHT9	HORVU6Hr1G073010.3	80.8	78.7	78.7	78.9

Supplementary table S2. Sequences of primers.

Name	Sequence (5’-3’)
qRT-PCR	
HvTHT1-F	CATGTCCAGCAGCTACCCGA
HvTHT1-R	GCCAAAGTCAACAAACGACTG
HvTHT2-F	CCTGTCCAGCAGTTACCCAG
HvTHT2-R	CCCGAAGTCGATAAACGACTG
HvTHT3-F	CAGATTGACTTCGGCGCC
HvTHT3-R	GAGTAGAAGGTCGCCGTGTC
HvTHT4-F	Used Hvtht3 F
HvTHT4-R	CACAAGTAACGCGTGTCATGATC
HvTHT5-F	GGCTCATGATCTTCATGCCA
HvTHT5-R	CAAGGGATTCCGGTGGTGTC
HvTHT6-F	GAACGCATCGACGACGAGTA
HvTHT6-R	CTATCCACCTCTGCGTCCG
HvTHT7/8-F	GGATGATGGTCTTCGTGCC
HvTHT7/8-R	GCAAATCTTGTTGAATGGCG
HvTHT9-F	GATGCTGCGTACATCCAGTC
HvTHT9-R	AGACTGCTGTCCACCTCCAG
ADP-F	GCTCTCCAACAACATTGCCAAC
ADP-R	GAGACATCCAGCATCATTCATTCC
Cloning of HvTht	
HvTHT8/pGST-F	CTGTTCCAGGGCCCGATGGAGGTTAACCACGCTGA
HvTHT8/pGST-R	TTCGGATCCCTCGAGTTACATCCTGGATGGGAGGA
HvTHT2/pGST-F	CTGTTCCAGGGCCCGATGGCAGTGATGGTGGAGAT
HvTHT2/pGST-R	TTCGGATCCCTCGAGTTAGTCCATTGAGTAGCAGATCTG
HvTHT7/pGST-F	CACCACCACATCGCCGACGGCCACGC
HvTHT7/pGST-R	GGCGATGTGGTGGTGGCATATCACGC
pGST inverse-F	CTCGAGGGATCCGAATTCAA
pGST inverse-R	CGGGCCCTGGAACAGAACTTC

Supplementary table S3. MRM conditions for triticamides and related compounds.

Compound	Precursor ion	Product ion	Cone voltage	Collision energy
	m/z	*m/z*	*V*	*eV*
Cinnamoyltryptamine	291.16	130.92	20	18
Cinnamoyl-9-hydroxy-8-oxotryptamine (**1**)	321.01	303.06	10	4
Cinnamoyl-8-oxotryptamine (**2**)	305.03	130.90	25	18
Cinnamoyl-(1*H*-Indol-3-yl)methylamine (**3**)	277.06	129.98	20	10
Tryptamine	161.02	143.93	16	10
8-Oxotryptamine	175.07	157.13	15	10
(1*H*-Indol-3-yl)methylamine	147.03	129.97	8	4
For detection of deuterium labeled phenylamides				
**1**-phenyl-*d*5	326.01	308.06	10	4
**2**-phenyl-*d*5	310.03	135.90	25	18
